# Ectopic Acromegaly Caused by Peripheral Ectopic Growth Hormone Hypersecretion: A Case Report and Literature Review

**DOI:** 10.7759/cureus.99840

**Published:** 2025-12-22

**Authors:** Nassim Ben Haj Slama, Imen Halloul, Malek Hadrich, Hamza Elfekih, Yosra Hasni

**Affiliations:** 1 Endocrinology, University Hospital of Farhat Hached, Sousse, TUN

**Keywords:** acromegaly, clivus, ectopic, growth hormone, pituitary adenoma

## Abstract

Acromegaly is most commonly caused by a growth hormone (GH)-secreting pituitary adenoma. In exceptionally rare circumstances, pituitary imaging fails to reveal an adenoma, thereby raising suspicion for ectopic acromegaly. Here, we report the case of a 70-year-old patient who presented for evaluation of headaches and glycemic control. Investigations confirmed ectopic acromegaly due to peripheral GH secretion. The source of GH secretion was a probable digestive neuroendocrine tumor (NET). Given the patient’s refusal of surgical intervention, treatment with a somatostatin analog was initiated, resulting in glycemic control consistent with the targets defined according to the patient’s age and comorbidities. Insulin-like growth factor 1 (IGF-1) levels subsequently normalized.

The diagnosis of GH-secreting ectopic acromegaly is based on correlating clinical manifestations with biochemical evidence of GH excess and appropriate imaging studies. Management is largely driven by surgical removal of the responsible lesion when possible, complemented by somatostatin analogs when indicated. The epidemiology and pertinent literature of this uncommon condition are discussed.

## Introduction

Acromegaly is an acquired endocrine disorder caused by excessive secretion of growth hormone (GH). It is characterized by typical facial, acral, skeletal, and systemic manifestations affecting multiple organ systems [1]. The estimated global prevalence ranges from 30 to 60 cases per million inhabitants [2].

GH secretion is normally regulated by the hypothalamic-pituitary axis: growth hormone-releasing hormone (GHRH) from the hypothalamus stimulates GH release from the pituitary, which in turn promotes production of insulin-like growth factor 1 (IGF‑1) by the liver and other tissues. This GH-IGF‑1 axis mediates the growth-promoting and metabolic effects of GH [3].

In most cases, GH excess originates from the pituitary gland, primarily due to a benign pituitary adenoma. However, in less than 1% of cases, no sellar lesion is detected, leading to the diagnosis of ectopic acromegaly [3]. This rare form of the disease results from extra-pituitary secretion of GHRH or, more rarely, GH itself [3].

The clinical manifestations and comorbidities associated with GH excess are similar regardless of the origin, but the therapeutic approach differs depending on whether the GH excess is ectopic or pituitary in nature [4]. Indeed, patients misdiagnosed with pituitary acromegaly may receive inappropriate treatment if their disease is actually due to a non-pituitary GH or GHRH-secreting tumor [4].

This paper presents the case of an elderly patient with acromegaly secondary to a digestive neuroendocrine tumor (NET) secreting GH, along with a literature review on ectopic GH secretion-related acromegaly.

## Case presentation

We report the case of a 70-year-old man evaluated for poorly controlled diabetes mellitus and headaches evolving over several months. His past medical history included long-standing hypertension (five years), well-controlled under treatment, and severe obstructive sleep apnea managed with continuous positive airway pressure. His diabetes, diagnosed 10 years earlier, was poorly controlled despite good adherence to metformin (2000 mg/day), glimepiride (6 mg/day), and lifestyle modifications.

On examination, he weighed 78 kg with a body mass index of 28 kg/m². Physical findings included a firm goiter and dysmorphic features such as thickened lips, coarse facial traits, a widened nasal base, prominent frontal wrinkles and bossing, and enlarged extremities with a reported shoe size increase in recent years.

Blood pressure was 130/80 mmHg. Ophthalmologic evaluation revealed no diabetic retinopathy or visual field defects.

Laboratory testing showed HbA1c of 10%. Given the clinical suspicion of acromegaly, serum IGF-1 was measured and found to be elevated at more than four times the upper limit of normal. An oral glucose tolerance test with GH suppression showed a GH nadir of 2.11 ng/mL (Table 1).

**Table 1:** Biological and hormonal investigations of our patient Abnormal indicators are highlighted in bold in the table.

Biology	Results	Normal values
Natremia	136	135 - 145 mmol/L
Kaliemia	3.8	3.5 - 5.0 mmol/L
Calcemia	2.3	2.1 - 2.6 mmol/L
Creatinine	75	50 - 90 µmol/L
Glycemia	10	3.9 - 53mmol/L
Glycated hemoglobin	10	4% - 5.6%
Insulin-like growth factor 1	850	<220 ng/mL
Growth hormone-releasing hormone	<60	<60 ng/mL
The lowest value of growth hormone during the oral glucose tolerance test	2.1	<1 ng/mL

**Figure 1:**
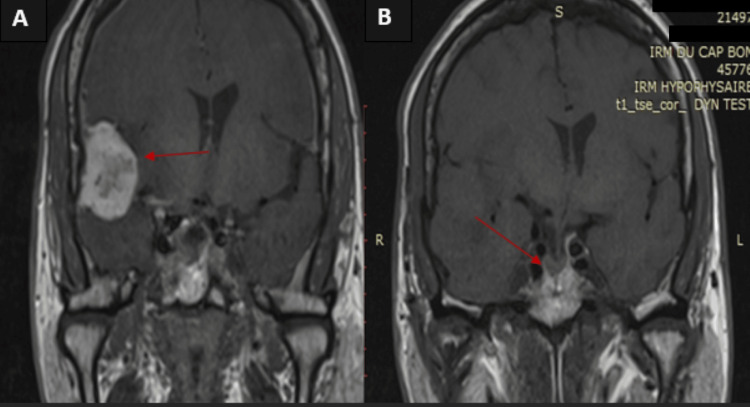
Hypothalamic–pituitary MRI of our patient Image 1A: Right temporoparietal extra-axial, dural-based mass consistent with meningioma, measuring 32 × 28 × 27 mm (red arrow), demonstrating significant mass effect with compression of the right lateral ventricle and associated leftward midline shift. Image 1B: Asymmetry of the pituitary gland, with increased thickness on the right, secondary to a mass effect on the midline, without any detectable adenoma (red arrow).

Pituitary MRI revealed a right temporoparietal space-occupying lesion consistent with a meningioma measuring 28 × 32 × 27 mm, exerting mass effect on the lateral ventricle. No pituitary adenoma was detected on any sequences (Figure 1).

A whole-body CT scan performed to search for a NET showed no suspicious lesions. Serum GHRH levels were within normal range (<60 ng/L).

Cervical ultrasound identified a 27 mm nodule in the left inferior thyroid lobe, classified as European Thyroid Imaging Reporting and Data System Category 3 (EU-TIRADS III).

**Figure 2:**
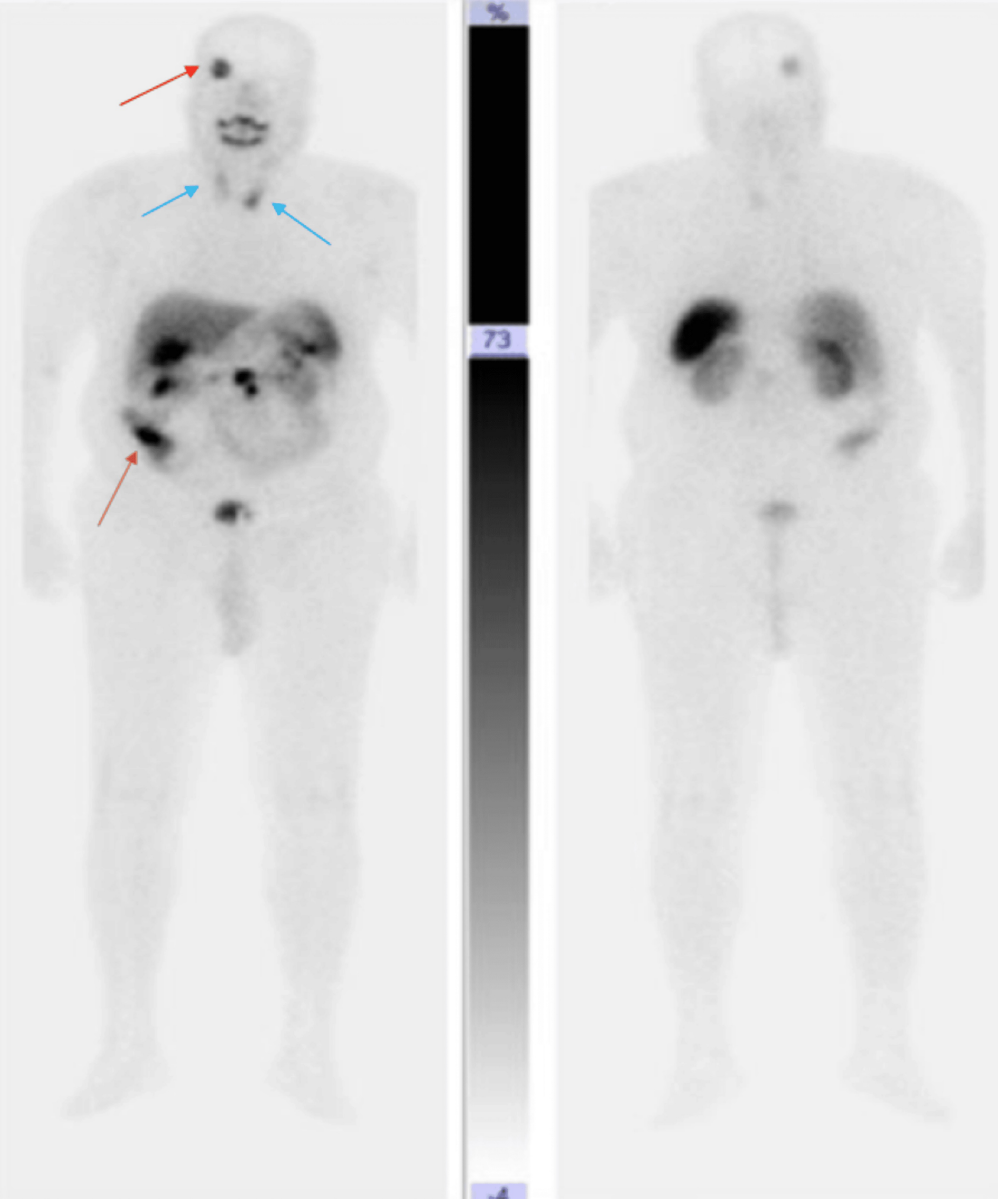
Octreoscan performed in our patient An octreoscan showing four sites of tracer uptake: uptake at the level of the right temporal meningioma (red arrow). No uptake was detected in the sella turcica or sphenoid sinus. Two sites of thyroid uptake, notable in the left inferior lobe (blue arrow). Intense uptake at the base of the cecum (brown arrow).

**Figure 3:**
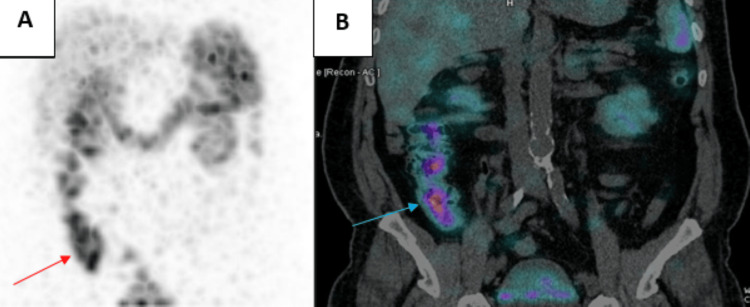
An octreoscan of our patient focused on the lesion located in the cecal base, combined with single-photon emission computed tomography coupled with computed tomography. A: Octreoscan showing intense tracer uptake at the base of the cecum (red arrow); B : Single-photon emission computed tomography coupled with computed tomography showing the intense uptake in the cecal base (blue arrow)

Somatostatin receptor scintigraphy revealed moderate radiotracer uptake in the right temporal meningioma, with no uptake in the pituitary region or sphenoid sinus. Two additional areas of mild uptake were noted in the thyroid gland, corresponding to the previously described nodules (Figure 2). A fourth focus of intense uptake was identified in the cecal base, persisting on delayed images (Figure 3).

Tumor markers, including calcitonin, carcinoembryonic antigen, and carbohydrate antigen 19-9, were within normal limits.

Colonoscopy showed a normal cecal base and terminal ileum, with a 4-mm cecal polyp resected and multiple sessile polyps in the sigmoid colon. Histopathological examination revealed a tubular-villous adenoma with low-grade dysplasia. Immunohistochemistry was negative for GH staining.

Due to worsening headaches, surgical resection of the right temporoparietal lesion was performed. Histopathological analysis confirmed a diagnosis of meningioma. Immunohistochemistry was also negative for GH staining.

For acromegaly management, long-acting somatostatin analog therapy (lanreotide 90 mg monthly) was initiated. Follow-up showed normalization of IGF-1 levels (179 ng/mL) and improved glycemic control (HbA1C 6.9%) on insulin glargine (8 IU) combined with metformin (2000 mg).

## Discussion

Case discussion

Acromegaly is a rare, chronic endocrine disorder with a potentially severe prognosis, caused by persistent GH hypersecretion leading to sustained elevation of circulating IGF-1 levels [5]. In more than 95% of cases, the etiology is a pituitary somatotroph adenoma [6-7]. However, in a minority of patients, acromegaly results from non-pituitary GH hypersecretion. These so-called ectopic forms arise from either excessive GHRH production or, more rarely, direct GH secretion by an extra-pituitary tumor [4].

Ectopic GHRH secretion is the most frequently reported mechanism and is typically associated with NETs, mainly of pancreatic or bronchial origin, though it has also been exceptionally described in other locations [4]. A literature review by Zendran et al. reported 127 cases of ectopic acromegaly due to GHRH hypersecretion [3], underscoring the exceptional rarity of this entity.

Even rarer are cases of acromegaly caused by ectopic GH secretion, with only a few documented in the literature [4]. Distinguishing these different forms of acromegaly is essential, not only for pathophysiological understanding but also for guiding therapeutic strategy [4].

According to Potorac et al., a small or hyperplastic pituitary gland, or the absence of a pituitary adenoma on MRI in a patient with acromegaly, suggests an ectopic source of GHRH secretion [8]. In our patient, the absence of both pituitary adenoma and hyperplasia, combined with normal GHRH levels, supported the diagnosis of GH-secreting ectopic acromegaly.

At this stage, thoracoabdominal CT imaging is recommended to localize the source of hormone secretion [9]. Most GH- or GHRH-secreting NETs are located in the lungs or pancreas [4]. In our case, conventional imaging failed to identify a neuroendocrine tumor.

Positron emission tomography (PET) using 68Ga-DOTA-TOC would have been the imaging modality of choice for detecting such tumors, owing to its high affinity for somatostatin receptor subtypes (SSTR) 2 and 5 and its excellent sensitivity and specificity [10].

In the absence of 68Ga-DOTA-TOC PET, the investigation was performed using somatostatin receptor scintigraphy. The Octreoscan has a sensitivity ranging from 75% to 100% and good specificity, although inferior to that of 68Ga-DOTA-TOC PET (50% vs 90.6%) [11].

In our patient, the octreoscan revealed four foci of tracer uptake, one of which corresponded to a physiological uptake in the meningioma. These tumors are known to express somatostatin receptors; however, to date, no case of GH-secreting meningioma has been reported in the literature.

Given the patient’s advanced age and high anesthetic risk, the initial therapeutic strategy was aimed at controlling acromegaly with somatostatin analogs while potentially benefiting from their antitumoral effect on the meningioma [12]. Nevertheless, due to the worsening mass effect from the lesion, surgical resection was ultimately required.

Histopathological and immunohistochemical examination of the resected specimen confirmed a meningioma with no GH immunoreactivity, consistent with previously published data.

Two additional foci of uptake were observed in the thyroid gland. Although cases of ACTH-secreting medullary thyroid carcinomas have been described [13], no GH secretion has ever been reported. Furthermore, investigations in our patient excluded the diagnosis of medullary thyroid carcinoma. The most likely diagnosis, therefore, remains that of a digestive NET, located in the cecum, secreting GH. Treatment with somatostatin analogs successfully controlled both acromegaly and the associated metabolic comorbidities.

Ectopic GH-secreting acromegaly

A classification proposed by Fainstein-Day et al. divides ectopic acromegaly into four distinct categories [4]: central ectopic GHRH secretion, peripheral ectopic GHRH secretion, central ectopic GH secretion, and peripheral ectopic GH secretion. In this review, we focus exclusively on ectopic GH secretion.

Central Ectopic GH-Secreting Acromegaly

Pathophysiology: The pituitary gland originates from Rathke’s pouch, an ectodermal evagination of the primitive stomodeum. During this complex process of migration and cellular differentiation, residual inclusions of pituitary tissue may persist along the path of invagination. Such remnants have been described at multiple sites within the sphenoidal region, including the mucoperiosteum of the vomerosphenoidal junction, the sphenoid bone, and, most commonly, the sphenoid sinus [14,15].

Topographically, the sphenoid sinus represents the most frequently reported site, likely due to its embryologic and anatomic proximity to the sella turcica [15]. The clivus is the second most common site, while more unusual localizations have been described in the cavernous sinus and suprasellar region [14].

Literature review of central ectopic GH-secreting acromegaly: Over the past 50 years, 19 cases of central ectopic GH-secreting acromegaly have been reported in the literature. 

Clinically, all patients presented with typical features of acromegaly, without specific symptoms related to the ectopic location of the adenoma. Tumor locations were predominantly the sphenoidal sinus (13 cases), followed by the clivus (six cases). Two patients presented concomitant sphenoidal and clival involvement. Guerrero et al. reported a suprasellar localization in a 31-year-old patient [16], while Mitsuya et al. described an ectopic somatotroph adenoma located within the cavernous sinus [17].

Functional imaging with somatostatin analog scintigraphy was performed in three patients, demonstrating pathological uptake in only one case.

In most cases, transsphenoidal surgery was the treatment of choice (17 cases), followed by somatostatin analog therapy used either alone (one case) or as adjuvant treatment (two cases). Dopamine agonists and radiotherapy were also used as adjuncts to surgery in some cases.

Histopathological and immunohistochemical analyses confirmed somatotroph pituitary adenomas in all cases, with co-expression of prolactin in eight patients.

Clinical outcomes were favorable in the majority of cases, with normalization of GH and IGF-1 levels in 11 patients and partial remission of acromegaly in six others.

All the cases of acromegaly due to central ectopic GH secretion are summarized in Table 2 [7, 14-31].

**Table 2:** Literature review of acromegaly due to ectopic central GH secretion GH: growth hormone; OGTT: oral glucose tolerance test; GHRH: growth hormone–releasing hormone; IGF-1: insulin-like growth factor-1; IHC: immunohistochemistry; PRL: prolactin; LH: luteinizing hormone; FSH: follicle-stimulating hormone; TSH: thyroid-stimulating hormone; SSTR: somatostatin receptor

Reference (author, year)	Age (years)	Gender	Tumor location	Size of the tumor (mm)	Imaging	Functional imaging	GH nadir during OGTT (ng/mL)	Preoperative GH (ng/mL)	Preoperative IGF-1 (ng/mL or ×ULN)	GHRH (ng/mL)	Therapeutic management	Outcome	Histology/Immunohistochemistry
Ferraz-Filho et al., 2012 [18]	30	F	Clivus and sphenoid sinus	30 x 20	Pituitary MRI	-		218	> 500	-	Surgery refused; Octreotide (dose not specified)	Tumor reduction, IGF-1 stabilization	-
Appel et al., 2012 [7]	50	F	Clivus	8 x 10	Pituitary MRI	-	4,8	-	937	-	Transsphenoidal surgery	Complete resection; IGF-1 normalization	Pituitary adenoma; IHC: GH+, PRL+, Ki-67: 3–4%, P53 <1%
Liu et al., 2013 [19]	56	M	Clivus	-	Pituitary MRI	-	-	200	-	-	Transsphenoidal surgery	Complete resection; IGF-1 normalization	Pituitary adenoma; IHC: GH+
Matsuno et al., 2001 [20]	51	M	Sphenoid sinus	-	Pituitary MRI	-	67	97	730	3,9	Transsphenoidal surgery	Complete resection; GH and IGF-1 reduction	Pituitary adenoma; IHC: GH+
Riccio et al., 2021 [14]	53	M	Clivus	8×5×9	Pituitary MRI	-	-	9,46	458	-	Transsphenoidal surgery	Complete resection; GH and IGF-1 normalization	Pituitary adenoma; IHC: GH+; Ki-67: 3%
Bhatoe et al., 2007 [21]	35	F	Clivus	-	Pituitary MRI	-	16	30,6	-	-	Transsphenoidal surgery	GH suppression during OGTT	Pituitary adenoma; IHC: GH+, PRL+, FSH+, LH+
Arzamendi et al., 2016 [22]	55	M	Sphenoid sinus	-	Pituitary MRI	Somatostatin receptor scintigraphy	6,37	16,7	560	7	Transsphenoidal surgery	Complete resection; GH and IGF-1 normalization	Pituitary adenoma; IHC: GH+, PRL+
Corenblum et al, 1979 [23]	59	M	Sphenoid sinus	-	Brain CT	-	31,9	46,8	-	-	Transsphenoidal surgery; Radiotherapy; Bromocriptine	Complete resection; GH reduction	Pituitary adenoma; IHC: GH+
Gondim et al., 2004 [24]	47	F	Sphenoid sinus	-	Pituitary MRI	-	-	97	862	-	Transsphenoidal surgery	Complete resection; GH and IGF-1 reduction	Pituitary adenoma; IHC: GH+
Guerrero et al., 2007 [16]	31	M	Suprasellar	-	Pituitary MRI	-	-	12,3	942	-	Right pterional craniotomy	IGF-1 normalization; GH suppression during OGTT	Pituitary adenoma; IHC: GH+, PRL+, rare ACTH+ cells; Ki-67: 1%
Madonna et al., 2001 [25]	60	F	Sphenoid sinus	-	Pituitary MRI	-	-	-	-	-	Transsphenoidal surgery	-	Pituitary adenoma; IHC: GH+, TSH+
Ramírez et al.. 2013 [15]	45	F	Sphenoid sinus	-	Pituitary MRI	Somatostatin receptor scintigraphy	2,5	7.7	920	-	Transsphenoidal surgery; Octreotide LAR 40 mg/month; Cabergoline 1.5 mg/week	Complete resection; GH and IGF-1 normalization	Pituitary adenoma; IHC: GH+, PRL+
Barry et al., 1982 [26]	a	M	Sphenoid sinus	-	Brain CT	-	26	33	3* LN	-	Transsphenoidal surgery; Bromocriptine 7.5 mg/day	IGF-1 normalization	Pituitary adenoma; IHC: GH+, PRL+
Kurowska et al, 2008 [27]	55	M	Sphenoid sinus	10	Pituitary MRI	Somatostatin receptor scintigraphy	4,3	4,3	615	-	Transsphenoidal surgery; Somatostatin analog	Complete resection; IGF-1 normalization	Pituitary adenoma; IHC: GH+, PRL+, SSTR3(+), SSTR5(+)
Hong et al., 2012 [28]	48	M	Sphenoid sinus	-	Pituitary MRI	-	Non freinée	18	1 120	-	Transsphenoidal surgery	Complete resection; IGF-1 normalization; GH suppression during OGTT	Pituitary adenoma; IHC: GH+
Hori et al., 2002 [29]	59	F	Sphenoid sinus	-	Pituitary MRI	-	Non freinée	14,54	-	-	Transsphenoidal surgery	Complete resection; GH reduction	Pituitary adenoma; IHC: GH+
Chan et al., 2005 [30]	58	F	Sphenoid sinus	-	Pituitary MRI	-	-	Élevée	Élevée	-	Transsphenoidal surgery; Radiotherapy	Complete resection; GH and IGF-1 normalization	Pituitary adenoma; IHC: GH+
Konar et al., 2013 [31]	51	F	Clivus and sphenoid sinus	-	Pituitary MRI	-	-	96	-	-	Transsphenoidal surgery; Radiotherapy	Incomplete resection; GH reduction	Pituitary adenoma; IHC: GH+
Mitsuya et al., 2004 [17]	55	F	Cavernous sinus	-	Pituitary MRI	-	-	133	731	-	Transsphenoidal surgery; Bromocriptine (dose not specified)	Incomplete resection; Persistent elevated IGF-1	Pituitary adenoma; IHC: GH+, PRL+

Acromegaly Due to Ectopic Peripheral GH Secretion

Pathophysiology: NETs comprise a heterogeneous group of tumors that can arise throughout the body and are characterized by their ability to secrete hormones in approximately 30% of cases [32]. These tumors exhibit a wide anatomo-topographic distribution.

The most common localization involves the gastrointestinal tract, accounting for more than 60% of cases predominantly within the midgut, followed by the foregut and hindgut to a lesser extent [32]. The lung represents the second most frequent site, encompassing over one-fifth of reported cases. More unusual localizations have been described in the cervicofacial region, thymus, thyroid, breast, skin, and genitourinary tract. In some instances, the tumor is discovered through metastatic lesions revealing an unidentified primary site. However, extra-digestive and extra-thoracic localizations remain exceptional [33].

The secretory profile of NETs varies according to their site of origin and may include insulin, somatostatin, glucagon, vasoactive intestinal peptide, and gastrin, among others [33]. In rare instances, NETs may induce acromegaly through ectopic secretion of GHRH. Even more exceptionally, acromegaly can result from direct ectopic production of GH itself [4].

Literature review of acromegaly due to peripheral ectopic GH secretion: Over the past 50 years, seven cases of ectopic acromegaly secondary to peripheral GH secretion have been reported in the literature. To our knowledge, our observation represents the eighth case.

No sex predominance was observed among the reported patients. Most patients presented with symptoms related to the primary tumor location, like dyspnea, chest pain, or cervical lymphadenopathy (five cases). Signs of acromegaly were also present in all patients.

The majority of NETs were located in the lung (three cases) or pancreas (two cases). An ovarian localization was reported by Ozkaya et al. [34]. Beuschlein et al., on the other hand, reported a case of ectopic GH secretion by a non-Hodgkin lymphoma [35]. Three cases presented with metastatic disease at diagnosis.

Two patients underwent functional imaging: the first underwent somatostatin receptor scintigraphy, which showed no abnormal tracer uptake [35]. The second was evaluated by 68Ga-DOTATOC PET/CT, demonstrating somatostatin receptor uptake consistent with a well-differentiated pulmonary NET [36].

Surgical resection of the tumor mass was the preferred therapeutic option in six cases. Chemotherapy alone was used in one case and as an adjuvant to surgery in two cases. Somatostatin analogs were administered to two patients in addition to surgery and chemotherapy.

Most patients experienced a favorable clinical outcome, with normalization of hormonal parameters (three cases) or partial improvement of acromegalic features (three cases). An unfavorable outcome was reported by Ezzat et al., with the patient’s death after three years of disease evolution [37].

All the cases of acromegaly due to peripheral ectopic GH secretion are summarized in Table 3 [34-40].

**Table 3:** Literature review of acromegaly caused by ectopic peripheral GH secretion GH: growth hormone; OGTT: oral glucose tolerance test; GHRH: growth hormone–releasing hormone; IGF-1: insulin-like growth factor-1; IHC: immunohistochemistry; MEN: multiple endocrine neoplasia; mRNA: messenger ribonucleic acid

Reference (author, year)	Age (years)	Gender	Tumor location	Size of the tumor (mm)	Imaging	Functional imaging	GH nadir during OGTT (ng/mL)	Preoperative GH (ng/mL)	Preoperative IGF-1 (ng/mL or ×ULN)	GHRH (ng/mL)	Therapeutic management	Outcome	Histology/IHC
Melmed et al., 1985 [38]	44	F	Pancreatic head	45	Abdominal CT, Pituitary CT	–	31	–	3 × ULN	<20	Pancreatic surgery	Complete resection; normalization of GH and IGF-1 levels	Pancreatic NET; IHC: GH+, GHRH–
Biswal et al., 2008 [39]	53	F	Lung	–	Chest X-ray, Chest CT, Bronchoscopy, Pituitary MRI	–	41.6	–	–	–	Left pneumonectomy	Complete resection; decrease in GH levels; symptoms regressed	Bronchial carcinoid NET; IHC: GH+, Chromogranin+
Krug et al., 2016 [36]	43	M	Lung	–	Pituitary MRI, Thoracic CT	Ga-68 DOTATOC PET-CT	10	Non-suppressed (exact value not specified)	1294	Not elevated	Right lobectomy	Complete resection; normalization of GH and IGF-1	Bronchial carcinoid NET; IHC: IGF-1+, Chromogranin+, Synaptophysin+, Ki-67 <2%, MEN1 gene–
Belloumi et al., 2021 [40]	55	M	Lung with hepatic, pulmonary, and bone metastases	80	Whole-body CT, Pituitary MRI	–	Elevated (pre-op, exact value not specified)	Not performed	Elevated (exact value not specified)	Not elevated	Left pneumonectomy; palliative chemotherapy (cisplatin + etoposide)	Post-chemotherapy: tumor stability but persistent acromegaly	Bronchial carcinoid NET; IHC: Chromogranin+, Synaptophysin+
Ozkaya et al., 2014 [34]	40	F	Ovary	95 × 80	Pituitary MRI, Abdominopelvic CT	–	29	30	1300	Not elevated	Ovarian surgery	Complete resection; GH suppression during OGTT	Ovarian carcinoid NET; IHC: GH+, GHRH–, Chromogranin+
Beuschlein et al., 2000 [35]	57	F	Abdominal (para-aortic, axillary/inguinal lymph nodes, hepatosplenomegaly)	–	Pituitary MRI, Abdominal CT	Somatostatin analogue scintigraphy	143	Non-suppressed (exact value not specified)	782	<0.02	Chemotherapy: cyclophosphamide, vincristine, doxorubicin, etoposide, prednisolone → relapse → fludarabine, mitoxantrone, dexamethasone; octreotide ineffective	Complete clinical and biochemical response after salvage chemotherapy; decrease in GH and IGF-1	Low-grade non-Hodgkin lymphoma; IHC: GH mRNA+, GHRH mRNA–, CD20+, CD79a+, somatostatin receptor–
Our case	70	M	Cecum	–	Pituitary MRI, Thoraco-abdominopelvic CT	Somatostatin analogue scintigraphy	2.1	–	850	<60	Lanreotide 90 mg monthly	Normalization of IGF-1 levels	–

Limitations of the case and literature review

In the case we report, the main consideration is the diagnostic uncertainty, as the tumor source of ectopic GH secretion could not be confirmed due to the patient’s preference for medical management. 68Ga-DOTATOC PET/CT, which offers higher sensitivity and specificity, was not available for this patient. Although less specific, the octreoscan remains of definite interest, particularly when 68Ga-DOTATOC PET/CT is not available.

Another limitation is the relatively short follow-up period of two years under somatostatin analog therapy, which may not capture the long-term evolution of acromegaly or potential tumor progression.

Regarding our literature review, it relied on previously reported cases that exhibit considerable heterogeneity in diagnostic methods, imaging modalities, and therapeutic approaches. Some older reports lacked detailed hormonal or imaging data, restricting the scope for comparative analysis. Furthermore, the extreme rarity of ectopic GH-secreting acromegaly limits the ability to draw broad conclusions or establish standardized diagnostic and treatment protocols.

## Conclusions

Acromegaly due to ectopic GH secretion is an exceptionally rare clinical entity. Confirmation of ectopic GH production relies on plasma GHRH measurement and/or histopathological evidence of GH secretion. In our case, GH secretion was ectopic, with the source not clearly identified, although a cecal origin is likely. The clinical presentation may vary depending on whether GH secretion is of peripheral or central origin.

Management primarily involves surgical excision of the tumor responsible for ectopic GH secretion. Somatostatin analog therapy can be used as an adjunctive treatment in cases of incomplete resection. They may also be employed, as in our case, when the patient declines surgery, and no identifiable ectopic GH-secreting tumor is found. Further case reports are needed to better characterize and advance understanding of this rare condition.
